# A feasibility study on integrating Generative AI and video conferencing into Indonesian recruitment processes

**DOI:** 10.3389/frai.2026.1784986

**Published:** 2026-05-13

**Authors:** Muhammad Ramaditya, Mohammad Ikhsan, Muhammad Firdaus Syawaludin Lubis, Corina D. Riantoputra

**Affiliations:** 1 Departement of Business Administration, Faculty of Administrative Science, Universitas Indonesia, Depok, Indonesia; 2 Electrical Engineering Department, Faculty of Engineering, Universitas Indonesia, Depok, Indonesia; 3 Department of Pyschology, Faculty of Psychology, Universitas, Indonesia, Depok, Indonesia

**Keywords:** feasibility study, Generative AI, HR technology, Indonesia, recruitment efficiency, video interview

## Abstract

The recruitment landscape in Indonesia faces a critical efficiency gap, with a global average time-to-hire of 44 days and a significant mismatch between labor supply and demand. This study evaluates “The Platform,” an innovative recruitment tool integrating Generative Artificial Intelligence (Gen-AI) and video conferencing to modernize interviews. To assess the project’s viability, we used a strategic management approach, including Political, Economic, Social, Technological, Environmental, and Legal (PESTEL) analysis to evaluate macro-environmental risks and opportunities; Porter’s Five Forces to analyze the competitive landscape and industrial profitability; and Strength, Weakness, Opportunity, and Threat (SWOT) analysis to identify internal strengths and weaknesses in the context of external factors. Furthermore, data from Focus Group Discussions (FGDs) with 58 Human Resources Managers were analyzed using a thematic approach to identify, assess, and map emerging Artificial Intelligence (AI) assumptions and user requirements. Findings reveal a green light for adoption, supported by high digital literacy in Indonesia, where a Serviceable Obtainable Market (SOM) is estimated at US$1.75 billion. Key success factors identified include the platform’s ability to conduct interviews in the local language and its alignment with national digital transformation goals. The study identifies culture lag and linguistic nuances as primary barriers to efficiency. Consequently, the study concludes that by addressing these identified efficiency gaps through robust data security and localized AI models, the platform is highly feasible and poised to revolutionize the Indonesian HR ecosystem.

## 1 Introduction

The Human Resources (HR) sector in Indonesia is currently seeing a huge amount of job seekers, thanks to the high number of Generation Z (Gen-Z) fresh graduates. According to Badan Pusat Statistik (2025), the unemployment rate in February 2025 was 4.76% or 7,278.31 million people. This condition is worsened by the effects of coronavirus disease 2019 (COVID-19) on the job market, in which the pandemic has reduced the chance of educated job seekers of getting jobs in the formal sector (Ridhwan et al., 2023). Even though approximately 18 million jobs have been created in 2025, the majority of them are in the informal sector (Ramadhan and Nugroho, 2025). The Nobel Prize-winning work of Peter Diamond, Dale Mortensen, and Christopher Pissarides made a collective contribution, known as the Diamond-Mortensen-Pissarides (DMP) or Search and Matching framework. In this view, the process of an unemployed worker finding a suitable job and a firm finding a qualified employee is inherently time-consuming and resource-intensive.

The global average time-to-hire now stands at approximately 44 days, with an average cost-per-hire of nearly US$4,700 (Deloitte, 2024). The recruitment funnel is extraordinarily leaky, requiring an average of 69 applicants to yield a single hire, a testament to profound inefficiencies in sourcing and selection (CareerPlug, 2024). Many Indonesian companies, especially small and medium-sized enterprises, still rely heavily on subjective “gut feelings” and personal connections rather than objective assessments of a candidate’s capabilities (Headhunter Indonesia, 2025). To combat these inefficiencies, Indonesian firms are increasingly reliant on HR technology, with the market valued at over US$6 billion in 2024 (Business Indonesia, 2025).

Bastida et al. (2025) stated that Artificial Intelligence (AI) increases operational efficiency, supports decision-making, and reduces costs. Regarding the use of Generative Artificial Intelligence (Gen-AI) in human resources management, Garcia and Kwok (2025) found that it automates tasks and creates new roles while still recommending the blend of AI and human power in HR processes, such as recruiting and training. This problem and the opportunities of the Indonesian job market are the core reasons for the creation of an AI-powered job interview platform for HR professionals that could help them process hundreds or even thousands of interviews per day. This feasibility study explores market, financial, and business conditions to determine the product’s Product-Market Fit (PMF). Please note that the recruitment process with Generative AI we are developing will be referred to as the recruitment process with Generative AI in this document.

The research gap identified in this study lies in the mismatch between labor supply and demand. At the same time, existing literature and tools address basic candidate filtering, but there is a lack of localized technology capable of handling the sheer scale of Indonesian applicants while maintaining high objectivity. Current AI tools in the market often suffer from “culture lag” or linguistic barriers, as many are developed for English-speaking contexts and fail to account for the nuances of Bahasa Indonesia and local dialects.

The developments in Human Resource Management (HRM) are increasingly pivoting toward Generative AI (Gen-AI) and “Agentic AI” to automate complex workflows. Recent studies suggest that Gen-AI can increase operational efficiency, support data-driven decision-making, and significantly reduce hiring costs (Do et al., 2025). Furthermore, industry projections indicate that by 2027, half of the companies utilizing Gen-AI will transition to Agentic AI systems capable of autonomous task execution and sophisticated candidate-job fit analysis. The integration of AI also aligns with broader environmental and social trends within Indonesia. For instance, the use of Gen-AI for text and task generation has been shown to emit significantly lower carbon emissions compared to human-led processes, supporting Indonesia’s Net Zero Emissions (NZE) goals. Additionally, social readiness is high, with approximately 83.6% of Indonesians reporting familiarity with AI technology, providing a fertile ground for the introduction of automated interview platforms.

Despite these advancements, a critical gap remains in AI’s ability to perform high-stakes behavioral analysis in local languages. While some local tools exist, they are often limited to audio-only formats, failing to capture essential non-verbal cues such as facial expressions. This limitation prevents HR professionals from fully trusting AI for final-stage interviews, necessitating a hybrid model that blends AI efficiency with human judgment. This study, therefore, proposes recruitment based on Generative AI as a state-of-the-art solution that addresses these gaps by offering facial expression analysis, fraud detection, and full support for Bahasa Indonesia. This development seeks to move beyond mere automation, fostering a “Human-AI Collaboration” that enhances the quality of hires while respecting the cultural and linguistic diversity of the Indonesian workforce.

## 2 Literature review

### 2.1 Resource-based view (RBV)

The resource-based view in strategic management is related to the opinion that the company’s internal resources are more important than external factors to achieve and maintain a competitive advantage [37, 38]. This approach is also determined by organizational performance, which is categorized into tangible, intangible, and corporate capabilities. According to the RBV, a set of tangible and intangible assets a company owns enhances its competitive advantage (Barney, 2001).

Tangible resources include physical assets such as land, buildings, factories, facilities, machinery, equipment, inventory, cash equivalents, marketable securities, receivables, technology, and organization. Intangible resources include human resources such as training, experience, intelligence, knowledge, expertise, innovation, reputation, and culture. Organizational resources or capabilities include customer service, product development, process innovation, systems, patents, brands, copyrights, and production processes (Benitez et al., 2022). Companies need to ensure their internal resources possess significant strengths that can be maintained and neutralize weaknesses. They also need to provide more support and encouragement, including developing employees’ abilities and skills (Ramaditya et al., 2022).

However, possessing unique resources alone is insufficient in dynamic business environments. Dynamic capabilities theory complements the RBV by emphasizing an organization’s ability to sense, seize, and reconfigure resources to remain relevant in the face of external changes (Teece, 2007; Kim and Song, 2014). In this study, AI adoption is positioned as a dynamic capability that enables both organizations and employees to adapt to technological change. AI adoption serves as a bridge between Draft Red Herring Prospectus (DHRP) and productivity outcomes, as human–AI collaboration enables employees to develop new competencies, reduce technostress, and enhance creativity.

### 2.2 Generative and agentic AI in HRM

Recent studies suggest that Gen-AI increases operational efficiency and supports data-driven decision-making. By 2027, half of the companies utilizing Gen-AI are expected to transition to “Agentic AI” systems capable of autonomous task execution. Implementation Gen-AI describes the extent to which employees and organizations integrate AI into work processes. AI automates routine tasks, supports personalized learning, and enhances creativity (Gupta and Bostrom, 2009; Do et al., 2025). Empirical studies show that AI adoption can increase productivity by 35.5% and job satisfaction by 20.6% (Shchekina, 2024).

### 2.3 Strategic environmental analysis

The study draws on Aguilar’s (1967) Political, Economic, Social, Technological, Environmental, and Legal (PESTEL) framework to assess macro-environmental influences and Porter’s (1979) Five Forces to analyze the competitive environment and business profitability. The environmental context captures external influences such as market competition, institutional dynamics, and ecosystem support.

Competitive Pressure describes the extent to which firms feel compelled to adopt innovations to maintain parity or achieve strategic advantage (Baig et al., 2019). In highly dynamic industries like telecommunications and financial services, competitive intensity accelerates HRA adoption as firms seek agility and credibility.

External support refers to assistance provided by technology vendors, consultants, or professional associations offering training, integration services, and post-implementation guidance (Hermann et al., 2024; Jayashree et al., 2022). From an institutional perspective, firms also respond to mimetic pressures, imitating peers to gain legitimacy (Kumar and Chen, 2024). Consequently, adoption decisions are not purely rational but socially constructed, reflecting both competitive and normative influences in the environment (Ansoff, 1958).

The organizational dimension emphasizes internal enablers that facilitate the use of Gen-AI. Top Management Support reflects the extent to which executives champion analytics initiatives, allocate adequate resources, and promote an AI culture (Shet et al., 2021). Leadership commitment legitimizes analytics as a strategic capability and fosters collaboration across departments. Organizational Readiness represents the availability of human expertise, IT infrastructure, and financial resources needed to adopt analytics successfully (Shahadat et al., 2023). In developing contexts, limited AI literacy and weak human resource data governance often constrain readiness (Wang et al., 2024; Espegren and Hugosson, 2025).

Prior empirical studies highlight that these internal factors play a supportive, though sometimes secondary, role compared to external environmental pressures (Su and Yang, 2023; Faiz et al., 2024; Badghish and Soomro, 2024). Thus, leadership engagement and organizational preparation remain necessary but insufficient without external facilitation.

## 3 Research method

The next stage is to identify processes or activities that either add to or change the business model. The purpose of this study is to explore the market, financial, and industrial factors that influence the development and, later, the marketization of the recruitment process with Generative AI in Indonesia. This study uses desk research to gather data from credible sources, including Badan Pusat Statistik, the official website of several relevant ministries, and scientific journal articles from reputable databases. The collected data are presented in tables and textual reports. To supplement desk research, a Focus Group Discussion (FGD) is also conducted to gain insights from HR professionals working at various Indonesian firms, who are members of an association of Indonesian HR professionals. The discussion centers on the HR sector’s readiness to use AI as an interviewer without human intervention and to weigh its advantages and disadvantages.

In preparation for this research, a resource analysis was carried out on 58 respondents, focusing on tangible and intangible resources and organizational capabilities. Respondents identified 10–15 internal factors for each component of internal analysis, which are relevant and important to the company and that also describe the company’s strengths and weaknesses. To validate the results, a Focus Group Discussion was carried out to identify, assess, and map various emerging Generative AI assumptions. Internal factors obtained from each component in the analysis resources are arranged based on the highest number stated by respondents. Assessment of the company was carried out through a questionnaire to 58 respondents with positions at the level of human resource managers in across industries in the private sector, such as banking, retail, transportation, IT services, education, and hospitality. The respondents’ criteria include those who have 5 years or more of experience in the field of human resources, good credentials, and an undergraduate degree or a master’s degree. The following qualifications include leaders at the managerial level and department heads who have a reasonably deep understanding and experience in various fields, specifically Human Resource Management. All the experts are given the chance to contribute by providing perspectives for PESTLE analysis and Strength, Weakness, Opportunity, and Threat (SWOT) analysis. Moreover, desk research was conducted using data from government sources (BPS, relevant ministries) and scientific databases. An FGD supplemented this with members of an Indonesian HR professional association to evaluate the advantages and disadvantages of AI interviewers. All Instruments were validated through the FGD stage, during which participants identified and mapped 10–15 relevant internal factors to describe organizational strengths and weaknesses. Qualitative data from the FGDs were analyzed by identifying internal factors and organizing them by the highest frequency of mention. Market size was calculated using standard Total Available Market (TAM), Serviceable Addressable Market (SAM), and Serviceable Obtainable Market (SOM) formulas based on firm counts and revenue thresholds.

## 4 Results

We analyze the existing market for the product using the PESTEL and Porter’s Five Forces frameworks formulated by Aguilar (1967) and Porter (1979), respectively. The former is a market analysis framework for assessing a business’s macro-environment, which influences its survival in the market, while the latter is a framework for analyzing the business’s competitive environment, in which it influences its profitability.

### 4.1 Macro and industry environment

The PESTEL analysis indicates that 83.6% of Indonesians are familiar with AI, providing a suitable premise for market entry. Porter’s Five Forces reveal that, while there are global competitors like interviewer.ai, they are limited to English-only services, giving local competitors a “low-threat” status. Table 1 presents PESTEL analysis—the purpose of assessing the macro-environmental factors (Political, Economic, Social, Technological, Environmental, and Legal) influencing the business. The key insight from these highlights is high social readiness (83.6% AI familiarity) and strong legal support through national digital strategies, though it notes risks, such as “culture lag.”

**Table 1:** Pestel analysis recruitment via Generative AI.

PESTEL analysis	
Political	The Net Zero Emissions (NZE) initiative is targeted to be achieved 41% by 2030 by the Indonesian government by integrating AI and Cloud Computing in various functions (Setiyono, 2022). This initiative is one of the key topics of the Golden Indonesia 2045 program, which aims to improve all aspects of Indonesians’ lives. This effort is supported by the regulations listed in the Legal section below.
Economic	The growth of Indonesia’s workforce is forcing companies to adopt strategies that enable them to process applications on a massive scale. This is especially important regarding job interviews, as they consume a lot of time per session.
Social	The bulk of the current workforce, Millennials (born 1981–1996) and Gen-Z (born 1997–2007), are familiar with digital technologies, especially AI. The use of Gen-AI, such as OpenAI’s ChatGPT and Google’s Gemini, is on the rise among these two generational cohorts. This is a suitable premise for introducing the recruitment process with Generative AI to the Indonesian market. This is supported by a report by the Katadata Insight Center (2025) that found approximately 83.6% of Indonesians are familiar with AI technology. This is a good start for the recruitment process with Generative AI, intended for HR professionals to conduct job interviews.
Technological	Regarding the effects of AI integration in HR management, Khan et al. (2024) found that it reduces hiring time and increases job–applicant matching accuracy. The technological infrastructure in Indonesia is improving with the construction of the Palapa Ring, a 12,000 km network that connects 57 cities, the construction of base transceiver stations at 1,600 points, and the launch of the Satria-1 satellite, which serves various functions (Kementerian Komunikasi dan Digital, 2024).
Environmental	Tomlinson et al. (2024) found that the carbon emissions from Gen-AI-generated texts are lower than those from human-generated texts. They found that a 250-word text generated by a Gen-AI emits approximately 3–4 g of carbon dioxide equivalent (CO_2_e) compared to 1,400 g if produced by a US national using a computer. Wang et al. (2024) also found that for every 1% increase in AI technology, there is a reduction of approximately 0.0025% in carbon emissions. These findings show that Gen-AI is approximately 1,000 times more efficient than humans. It provides a supportive argument for using Gen-AI in conducting job interviews, since the effect is parallel across all types of activities involving Gen-AI.
Legal	The Indonesian national government regulates the use of AI by enacting these laws: Undang Undang No. 1 Tahun 2024 (JDIH Badan Pemeriksa Keuangan, 2024) Peraturan Pemerintah No. 71 Tahun 2019 (JDIH Badan Pemeriksa Keuangan, 2019) Surat Edaran Menteri Komunikasi dan Informatika No. 9 Th. 2023 (JDIH Kemkomdigi, 2023) National Strategy of Artificial Intelligence 2020–2045 (Kementerian Komunikasi dan Digital, 2024) National Strategy of Digital Economy (Kementerian Komunikasi dan Digital, 2024)

Table 2 presents Porter’s Five Forces—the purpose of analyzing the competitive environment and industry profitability. The key insight is that the competition is currently low; English-only services limit global tools like interviewer.ai, whereas this platform supports Bahasa Indonesia.

**Table 2:** Porter’s five forces recruitment with Generative AI.

Porter’s five forces	
New entrants	Recruitment via AI has launched its AI-powered video interview tool into the market. However, it is currently only available in an audio-only format. The inability to analyze candidates’ facial expressions and detect fraud is the core reason the recruitment process with Generative AI was created.
Substitute product(s)	*interviewer.ai* is the closest to being a competitor of the recruitment process with Generative AI. However, all services are offered only in English, and they cannot be used for the *final* interview, which requires HR professionals to physically interview candidates after conducting preliminary interviews and deciding which candidates to interview.
Suppliers’ power	OpenAI’s GPT-4o Mini will be the powerhouse of the recruitment process with Generative AI. This LLM provides the delta transcripts and responses in real time. This means the recruitment process with Generative AI is fully dependent on OpenAI to run its video interview service. Docker (http://www.docker.com) will provide the application container for facial expression analysis and fraud detection in the recruitment process, enabling Generative AI to run seamlessly.
Buyers’ power	The recruitment process for Generative AI’s buyers will involve HR professionals from various Indonesian firms. They already have a set of standards or requirements that the recruitment process with Generative AI must meet if it wants to partner with them. One thing to be aware of is that they are free to choose whichever platform meets *all* their requirements. Large firms often have their own compliance standards and demand customization and integration with their core systems. Furthermore, the Indonesian government is rather strict when dealing with legal affairs, so the recruitment process with Generative AI must meet all legal requirements to be approved for commercialization. In conclusion, the recruitment process with Generative AI must absolutely adhere to the regulations enforced by the Indonesian government.
Competition	As of the time of this writing, the recruitment process with Generative AI has no local competitor. Only *interviewer.ai* poses a competition threat to it, but considering its limitations as described above, it will be a low-threat potential competitor to the recruitment process with Generative AI. It must utilize its novelty/innovation advantage to leverage its success, since there is still no local competitor.

#### 4.1.1 Potential market analysis

The potential market for the recruitment processes with Generative AI is assessed based on market size and market growth aspects. We used the Strength, Weakness, Opportunity, and Threat (SWOT) framework proposed by Humphrey (2005) to analyze the recruitment process with Generative AI’s potential. Table 3 presents SWOT analysis—the purpose of identifying internal Strengths/Weaknesses and external Opportunities/Threats. Key insight is that the AI recruitment platform’s ability to detect fraud and analyze facial expressions is a core strength, while “robotic” communication remains a weakness.

**Table 3:** SWOT analysis recruitment with Generative AI.

Strengths (S)	Weaknesses (W)
It has facial expression analysis and fraud detection systems, which are crucial to decide whether to hire a candidate. It eliminates the need for the presence of a human interviewer, which saves time and energy for the whole recruitment process. It is designed to conduct interviews in Bahasa Indonesia. This opens an opportunity for firms and candidates who want to conduct job interviews in the language.	The AI’s communication style is still robotic. There is still a need for a usability test on actual job seekers.

Opportunities (O)	Threats (T)
Recruitment process with Generative AI’s AI interviewer can be designed to be more human-like. Demand from large Indonesian firms, which number approximately 6,500 firms. Market growth opportunity is high, with the recruitment process with Generative AI as a pioneer in its industry. An English-language version could be added, so that firms that want to conduct interviews in English could use the recruitment process with Generative AI.	Culture lag in using an AI-powered video interview platform can potentially occur. Legal requirements related to market launch could hinder its development. The threat of unmitigated risks that can set its market launch back still exists. Resistance from HR professionals and a portion of the general public.

Determining the market size for the recruitment process with Generative AI requires breaking the market down into Total Available Market (TAM), Serviceable Addressable Market (SAM), and Serviceable Obtainable Market (SOM). Table 4 presents the estimated numbers of firms in Indonesia.

**Table 4:** Estimated number of firms in Indonesia.

Information	Total Firms
All firms	3,441,481 firms
Medium-sized firms	115,943 firms
Large-sized firms	52,662 firms

Below are the formulas for calculating TAM, SAM, and SOM in monetary terms.


$$\begin{array}{l} \mathrm{TAM}=\text{Total}\;\;\text{number of customers}\\ \times \text{Average}\;\;\text{annual revenue}\;\mathrm{per}\;\text{customer} \end{array}$$

$$\begin{array}{l} \mathrm{SAM}=\text{Total number of customers}\times \%\text{Serviceable customers}\\ \times \text{Average}\;\;\text{revenue}\;\mathrm{per}\;\text{customer} \end{array}$$

$$\begin{array}{l} \mathrm{SOM}=\text{Total}\;\;\text{number of customers}\times \%\text{Serviceable customers}\\ \times \%\text{Capturable market}\times \text{Average}\;\;\text{revenue}\;\mathrm{per}\;\text{customer} \end{array}$$

Table 4 presents the estimated number of firms in Indonesia: 3.4 million total firms, with a focus on 115,943 medium and 52,662 large firms. The market size calculations (TAM, SAM, and SOM) are TAM (total available market): US$175.4 billion; Serviceable Addressable Market (SAM): US$35.1 billion; and Serviceable Obtainable Market (SOM): US$1.75 billion. This represents the realistic initial target for the AI recruitment platform in Indonesia. Since the number of firms in each category is known, the average annual revenue per customer should be determined. The number is based on UU No. 20 Th. 2008 (JDIH Badan Pemeriksa Keuangan, 2008). Since the annual revenue threshold for large firms is only specified as more than Indonesian Rupiah (IDR50) million (Pemerintah Daerah Kabupaten Sumbawa Barat, 2020), the lower threshold is used to calculate TAM, SAM, and SOM.

TAM


$$\begin{array}{l} \mathrm{TAM}\;(\text{medium}\;\;\text{firms})=115,943\times \mathrm{US}\text{\$}150,060\\ =\mathrm{US}\text{\$}17,398,406,580 \end{array}$$

$$\begin{array}{l} \mathrm{TAM}\;(\text{large}\;\;\text{firms})=52,662\times \mathrm{US}\text{\$}3,001,200\\ =\mathrm{US}\text{\$}158,049,194,400 \end{array}$$

$$\begin{array}{l} \mathrm{TAM}=\mathrm{US}\text{\$}17,398,406,580+\mathrm{US}\text{\$}158,049,194,400\\ =\mathrm{US}\text{\$}175,447,600,980 \end{array}$$

SAM


$$\begin{array}{l} \mathrm{SAM}\;(\text{medium}\;\;\text{firms})=115,943\times 0.20\times \mathrm{US}\text{\$}150,060\\ =\mathrm{US}\text{\$}3,479,681,316 \end{array}$$

$$\begin{array}{l} \mathrm{SAM}\;(\text{large}\;\;\text{firms})=52,662\times 0.20\times \mathrm{US}\text{\$}3,001,200\\ =\mathrm{US}\text{\$}31,609,838,880 \end{array}$$

$$\begin{array}{l} \mathrm{SAM}=\mathrm{US}\text{\$}3,479,681,316+\mathrm{US}\text{\$}31,609,838,880\\ =\mathrm{US}\text{\$}35,089,520,196 \end{array}$$

SOM


$$\begin{array}{l} \mathrm{SOM}\;(\text{medium}\;\;\text{firms})=115,943\times 0.10\times 0.10\times \mathrm{US}\text{\$}150,060\\ =\mathrm{US}\text{\$}173,984,066 \end{array}$$

$$\begin{array}{l} \mathrm{SOM}\;(\text{large}\;\;\text{firms})=52,662\times 0.10\times 0.10\times \mathrm{US}\text{\$}3,001,200\\ =\mathrm{US}\text{\$}1,580,491,944 \end{array}$$

$$\begin{array}{l} \mathrm{SOM}=\mathrm{US}\text{\$}173,984,066+\mathrm{US}\text{\$}1,580,491,944\\ =\mathrm{US}\text{\$}1,754,476,010 \end{array}$$

Based on the calculations above, here are the TAM, SAM, and SOM of the recruitment process with Generative AI. The commercial life cycle of the recruitment process with Generative AI is divided into pre-market, market entry, and retention. Each phase has its own set of activities that the developers of the recruitment process with Generative AI must conduct to move on to the next phase. Here are the aforementioned activities.

Table 5 presents the pre-market (legal/usability testing), market entry (sales/social selling), and retention (24/7 support/surveys) phases to make the AI recruitment platform successful in the Indonesian business.

**Table 5:** Commercial life cycle.

Life cycle phases	Focus area(s)	Activities
Pre-market	Legal Marketing Public relations (PR) Business development	Completing all legal requirements Evangelizing the recruitment process with Generative AI to the public and potential customers Preparing a public presence of the recruitment process with Generative AI Conducting usability testing to identify new features to add and features to eliminate
Market entry	Sales Marketing Business development	Promotional activities through social media, social selling, and other channels Conducting rigorous sales through various channels Continuing to add new features and eliminate unpopular features
Retention	Operations Aftersales	Conducting routine checkups of the recruitment process with Generative AI to ensure a seamless operation Eliminating technical errors that may occur during its operation Providing aftersales services such as 24/7 customer service support, guides for a successful use of the recruitment process with Generative AI, product use surveys, etc.

Since the market size has been determined, it is now possible to conduct a market growth analysis. The numbers of SAM and SOM are US$35,089,520,196 and US$3,508,952,020, respectively, based on the number of medium- and large-sized firms and each firm’s annual revenue, as per the act mentioned above. The market target characteristics are as follows: Indonesian medium- and large-sized firms that operate in all provinces and cities, process hundreds to thousands of candidate interviews each day or month, and are seeking a digital platform to minimize overall recruitment time.

To calculate market growth, the growth of the number of medium- and large-sized firms must also be taken into account. This growth is assumed to be exponential, meaning there are no dips in the growth trend. In the context of recruitment via an AI platform, it is best to choose the Product Development strategy, as it aims to enable AI-driven video interviews rather than the audio-only format currently employed. Here are the tactics that should be employed to achieve success which is establish additional partnerships with medium- and large-sized Indonesian firms using personal connections with HR professionals; conduct rigorous usability testing with potential users/customers to ensure the right features (i.e., features that customers need or want) are added; set competitive prices for the video interview service to generate sufficient revenue and profit; and create an opportunity for potential customers to ask questions or give feedback on Recruitment process with Generative AI.

#### 4.1.2 Industrial analysis

The recruitment process with Generative AI operates in the technology sector of the HR technology industry. It is a novel innovation because it is the first recruiting technology to use an AI model. The HR technology industry in Indonesia is experiencing an uptick in the use of AI in recruiting and talent management processes (Insight Sinergi Talenta, 2025). They also stated that AI speeds up the candidate filtering process, helps professionals analyze employee data, and provides predictive analyses to assist in decision-making processes. These situations support the need for HR professionals in Indonesia to use generative AI in the recruitment process to make their recruitment work more efficient. Here are some general industry trends that are predicted by Deloitte (2025):

1. Investments and market conditions are getting better, which leverages the HR technology industry.
2. Agentic AI, a type of AI that is autonomous in conducting decision-making and performing tasks, is automating workflows that are deemed complex by humans. In 2027, approximately 50% of companies that use Gen-AI will launch Agentic AI.
3. Candidates’ skills will be assessed using Agentic AI that could deliver better analyses and increase candidate-job fit.
4. Task management will shift focus from job titles to task descriptions to produce better workforce alignment.
5. Personalization is getting more attention in HR technologies.
6. Microcultures (i.e., the unique culture of each department, office branch, and level) are getting more powerful in shaping workforce experience by shaping how employees behave and act in the workplace according to their teams’ cultures.

These findings indicate that the HR tech industry is focusing on personalization and individualism in formulating organizational cultures. The personalization of HR technologies is the main point of discussion in this study, and the recruitment process with Generative AI can learn several lessons from these findings. In Indonesia, the HR tech industry is experiencing the same phenomena described above. Business Indonesia (2025) reported that digitalization in HR is a manifestation of the popular demand for hybrid work arrangements, project-based jobs, and scalability of business operations. Government incentives are also in place for firms that implement digital HR practices. The transition to green practices is also underway, with approximately 20,000 employees projected to be in various green economy roles by 2029 and the creation of 10 standards for green work (Ramaditya et al., 2023).

Several academics have studied the ever-increasing importance of AI in the HR tech industry. For example, da Silva et al. (2022) found, through their literature review, that AI is used in the recruitment processes of various firms and non-profit organizations alike, reducing data inaccuracies and automating resume screening. Li and Cheng (2025) in their literature study found that ChatGPT and other Generative AI models help organizations in creating equity and reducing inequities during recruitment. Green Human Resource Management (GHRM), combined with the use of AI and the Internet of Things (IoT), leverages environmental management systems (Ahmad et al., 2025). Nishanti et al. (2025) pointed out that the adoption of AI in Human Resource Management surged after the COVID-19 pandemic, and that more studies on the topic have emerged in the education and social sciences disciplines (Mujianto et al., 2025).

#### 4.1.3 Financial analysis

This section contains several pricing schemes and a cost projection for the recruitment process with Generative AI. The pricing strategy is based on the current prices of the audio-only video interview feature. The cost projection is created using data from the Research and Development (R&D) budget plan document and assumptions for the marketing and operations costs (Brealey et al., 2020). Learning from OpenAI’s ChatGPT and other paid models, it is a good idea to create three pricing schemes. Each pricing scheme must include a limit on the number of interviews allowed in a given period.

The AI model’s ability to analyze the candidate’s transcript of each interview should be included in all schemes, as it is a core feature of the recruitment process with Generative AI, inherited from the current recruitment via the AI audio-only model. Meanwhile, the video analysis features such as facial expression analysis and fraud detection by lip movements should be limited to the least to most expensive schemes. It is logically the least expensive feature that offers the fewest video-specific features. A piece of information to note is the maximum recorded simultaneous sessions, which were 1,500 interviews. Here are the three pricing schemes and their features. Table 6 presents the pricing schemes—this study proposes three tiers: Basic (IDR6,000/interview), Pro (IDR8,0000/interview), and Max (IDR10,000/interview). The key insight is that advanced features like “interview jockey” and long-term cloud storage are reserved for higher tiers.

**Table 6:** Estimation of pricing scheme of recruitment via Generative AI.

Pricing scheme	Price	Features
Basic	IDR6,000/interview	Maximum 1,000 interviews per period Interview duration maximum 30 min Video transcript (PDF file) Facial expression analysis Fraud detection: Lip sync Script reading Candidate interview performance assessment (PDF file) Cloud storage up to 30 days
Pro	IDR8,000/interview	Maximum 5,000 interviews per period Interview duration maximum 60 min Video transcript (PDF file) Facial expression analysis Fraud detection: Lip sync Script reading Interview jockey Interview analysis (PDF file) Cloud storage up to 90 days
Max	IDR10,000/interview	Maximum 10,000 interviews per period Interview duration maximum 120 min Video transcript (PDF file) Facial expression analysis Fraud detection: Lip sync Script reading Interview jockey Pre-recorded answers Candidate interview performance assessment (PDF file)

The price of each scheme is determined by several factors. The size of SAM is among the factors since it indicates the total number of firms that have the potential to become involved in the recruitment process with customers with Generative AI. The second factor is the average price of such services in the HR tech industry, which is approximately US$100–150 (approximately IDR1.5–2.5 million). The cost projection for the recruitment process with Generative AI is based on assumptions about what costs could possibly occur once the recruitment process with Generative AI is commercialized.

The collaboration aims to integrate recruitment via Generative AI capabilities directly into the job-seeking ecosystem, enhancing candidate readiness, improving employer hiring efficiency, and generating new monetization streams for both parties. This collaboration enhanced strategic partnership throughout the Co-opetition Framework, which was adapted from Ritala and Hurmelinna‐Laukkanen (2012). The collaboration between recruitment via Generative AI and leading job platforms has strong potential to be integrated with the Top 20 Job Posting Sites in Business Indonesia (2025), including *JobStreet Indonesia, Kalibrr, Indeed, Glints,* *http://www.Karir.com**, TopKarir, Tech in Asia Jobs, Lokerindo.id, LinkedIn, KarirLab, KompasKarier, OLX Lowongan Kerja,* *http://www.Jobindo.com**, 9cv9, Snaphunt, Grabjobs,* *http://www.Recruit.net**, The Jakarta Post Jobs, Jooble,* and *KitaLulus.* This opportunity arises because, among active job posting platforms in Indonesia, approximately 70–80% still lack sufficient AI interview technology. This means recruitment via AI has a substantial partnership potential with approximately 7–20 platforms. Moreover, collaboration between AI-based recruitment and job-seeking platforms can reduce the candidate–employer mismatch during the screening stage by 20–40%, according to benchmark studies on AI-based assessment implementations by LinkedIn (2023).

The strategic plan for recruitment via AI presents a comprehensive and methodically structured approach to market adoption, leveraging the well-established “5A” customer journey framework (Aware, Appeal, Ask, Act, Advocate) proposed by Kotler et al. (2017) in their book *Marketing 4.0: Moving from Traditional to Digital*.

- The strategy initiates with the “Aware” phase, aiming to build foundational market presence and credibility by publishing pilot success stories and engaging with professional HR associations to establish authority.
- This seamlessly transitions into the “Appeal” stage, where the focus shifts to creating a compelling value proposition by quantifying concrete benefits, such as a 50% cost reduction and 40% faster time-to-hire, while mitigating perceived risks through free trials to lower the barrier to entry.
- The “Ask” and “Act” phases are designed to convert interest into commitment; the plan strategically targets high-value clients in the private sector and addresses critical enterprise concerns such as data security and system integration (Applicant Tracking System [ATS]) to ensure a smooth transition and long-term viability.
- Finally, the strategy culminates in the “Advocate” phase, which aims to transform satisfied clients into brand promoters through case studies and community engagement, effectively creating a sustainable growth loop built on trust and proven results.

As with all other projects, this project to develop a recruitment process with Generative AI has its risks. Identifying and mitigating risks can be a lifesaver for the project, since unmitigated risks can turn the whole project upside down if the project developers do not know how to mitigate those risks. The risks are divided into external and internal risks, with the former analyzed using the PESTEL framework below. Table 7 presents the risk strategy, which details external threats like “technological resistance” from HR professionals who fear job loss. This study recommends positioning the AI as a “digital assistant” rather than a competitor.

**Table 7:** Risk strategy.

PESTEL element	Risk	Risk level	Mitigation strategy
Political	New or revised acts that restrict the use of AI in processing personal information	High	Establish a connection with politicians and parties to secure the survival of the recruitment process with Generative AI. Maintain compliance with regulations and government institutions.
Economic	Inflation that increases production costs, particularly the costs of operating the AI model and cloud computing	Medium	Adopt a lean firm structure to minimize costs. Include the inflation rate in budgets. Create fixed-rate contracts to avoid getting charged more due to inflation. Set flexible prices that adjust to inflation and deflation.
	Deflation that reduces the number of purchases	Medium	
Social	Culture shock experienced by HR professionals who were not used to using AI in conducting candidate interviews	Medium	Conduct free tests with HR professionals to habituate them with the recruitment process with Generative AI. Inform potential customers of the safety and compliance of the recruitment process with Generative AI. Present the benefits of using the recruitment process with Generative AI in a persuasive manner.
	Technological resistance from HR professionals who fear losing their jobs	High	Position the recruitment process with Generative AI as a “digital assistant” rather than a “digital competitor/disruptor” to the HR professionals. Educate the HR professionals on the benefits of using AI in job interviews.
	Different technological literacy levels across companies	Medium	Provide educational programs on how to use the recruitment process with Generative AI effectively. Design a UX that is easily understandable for the lowest-literate customers.
Environmental	Disasters that disrupt the recruitment process with Generative AI’s data centers	Medium	Establish disaster recovery protocols. Create data backups in case of emergencies.
Technological	System errors during the recruitment process with Generative AI’s operation	High	Create error protocols. Utilize adequate redundancy in the cloud computing system.
	Inaccurate scoring due to unintentional mistakes by the AI model	High	Train the AI model to recognize variations in facial expressions, figures of speech, etc. Conduct routine audits on the AI model’s performance to identify and solve scoring issues. Employ human assessors to double-check the assessment results made by the AI model. Create robust training materials to minimize this type of error.
Legal	Oversighted non-compliance with related regulations	Medium	Implement end-to-end encryption that could prevent high-level data breach attempts. Conduct data safety assessments regularly. Create a data protection corporate rule that contains a set of protocols that deal with data protection and safety management.

In addition to external risks, internal risks should be identified and mitigated. Therefore, here is a list of potential internal risks and their mitigation strategies. The internal risk mitigation strategy based on Table 8 is directly linked to the study’s RBV framework. For example, risks such as “inaccurate assessments” are mitigated by training the AI model to recognize variations in facial expressions—an intangible organizational capability. Financial risks like “insufficient funding” are managed through accurate cost calculations and digital accounting tools.

**Table 8:** Internal risk mitigation strategy.

Area of operation	Risk	Risk level	Mitigation strategy
Financial	Insufficient funding that could lead to bankruptcy	High	Calculate all costs as accurately and factually as possible. Utilize a cloud-based digital accounting tool that could produce more accurate results compared to human-made ones.
	Inaccuracies in financial forecasts	High	
Marketing	Inadequate brand awareness	High	Establish a digital presence on popular social media platforms (e.g., Instagram and TikTok). Create effective marketing content to attract an audience and therefore potential customers.
	Falling into irrelevance due to ineffective search engine optimization (SEO)	Medium	Utilize keywords commonly associated with AI, job interviews, and digital HR. Learn about popular keywords on each social media platform to better design the SEO practices.
Operations	Siloed operations of each department	Medium	Establish a transparent organizational culture to prevent siloed operations. Conduct routine interdepartmental events to remove interaction barriers and negative competition between departments.
	Bugs during interviews	High	Optimizing the AI model and API to minimize bugs. Conduct quality inspections routinely.
	Delays in uploading video interview data to the server	High	Enable autoscaling for high and low traffic. Ensure fast internet uploading speeds at all times.
	Inaccurate assessments of candidates’ facial features	High	Train the AI model to recognize variations in the meaning of facial expressions.
Human resources	Low candidate numbers in the first 1–3 years	High	Provide practical benefits to attract more candidates. Create engaging and effective recruitment ads on various advertising platforms.
	Difficulty in recruiting highly skilled individuals	Medium	Offer flexible work arrangements to attract those who prefer remote and hybrid work. Research common benefits and compensations that those professionals commonly demand.
Public and customer relations	Misalignment between the message and the values	High	Unify all departments under the same vision and value proposition. Create clear guidelines on how to position the recruitment process with Generative AI in the public eye to prevent wrong messages delivered by employees.
	Negative reviews and comments by dissatisfied customers	High	Answer any questions from potential customers as clearly as possible. Provide a clear description of the recruitment process with Generative AI on its website.
	Fake negative reviews from possible competitors	Medium	Regularly review all customer feedback and reviews to identify and delete such fake reviews. Address genuine negative reviews to show the difference between genuine and fake negative reviews to customers and the public alike. Eliminate malicious bot activities using an automated bot detection and elimination system.

## 5 Discussion

The current recruitment landscape in Indonesia is defined by a significant volume-to-efficiency gap. With over 7.27 million unemployed individuals and a massive influx of Gen-Z graduates, HR departments are overwhelmed by the sheer scale of applications. Data indicates that for every hire, recruiters must navigate dozens of profiles, often resulting in a “time-to-hire” that exceeds global benchmarks. This bottleneck not only delays corporate productivity but also creates a frustrating experience for candidates who are left in a state of professional limbo. To bridge this gap, “The HR-AI Platform” introduces a sophisticated Gen-AI and Video Conferencing solution designed to modernize the initial screening process. By automating the preliminary interview phase, the system provides an objective, data-driven assessment of candidate capabilities. This is not just about recording a video; it is about using advanced algorithms to interpret non-verbal cues and verbal proficiency in real time, allowing recruiters to focus their energy on high-potential talent rather than on administrative filtering.

Market analysis reveals a fertile ground for such innovation. With Indonesia’s Internet penetration and smartphone usage at all-time highs, the digital literacy required to navigate AI platforms is already widespread. Our study shows that over 83% of the population is familiar with AI tools, and the government’s “Golden Indonesia 2045” vision further incentivizes the adoption of “Agentic AI.” This favorable environment suggests that the primary barrier is no longer the technology itself, but its strategic implementation. Financial projections underscore the massive scale of this opportunity. The Serviceable Obtainable Market (SOM) for such a platform in Indonesia is estimated at approximately US$1.75 billion, driven by the needs of over 6,500 large-scale enterprises and thousands of growing SMEs. By implementing a tiered subscription model ranging from “Trial” to “Business” and “Enterprise” packages. The recruitment process with Generative AI can cater to a diverse range of budgets while maintaining a sustainable revenue stream. However, the Focus Group Discussions with HR professionals highlighted that success is predicated on trust and security. Participants were vocal about the risks associated with personal data; consequently, the recruitment process with Generative AI must employ high-level encryption and robust firewall systems. In an era where data breaches are a constant threat, guaranteeing the privacy of candidate information is not just a feature; it is a foundational requirement for market entry.

Beyond security, the FGD participants emphasized the “Indonesian context.” A generic global AI might struggle with the nuances of local communication. Therefore, a critical recommendation is the development of a localized linguistic engine. This engine must be capable of distinguishing among regional dialects, intonations, and slang to ensure that a candidate from Medan or Surabaya is evaluated as fairly as one from Jakarta, thereby eliminating inherent geographic biases. Another strategic pivot identified was the need for flexibility. HR professionals expressed a desire to move away from “black-box” assessments. They want the ability to customize assessment metrics to match their specific organizational culture and job requirements. By allowing firms to adjust the weight of different soft skills or technical competencies, the recruitment process with Generative AI transforms from a rigid software tool into a versatile HR partner that respects the unique identity of every firm. The social and cultural implications of AI cannot be ignored. There is a palpable fear among HR staff regarding job displacement. To mitigate this, the narration of the recruitment process with Generative AI must focus on “Human-AI Collaboration.” The AI identifies the “unique thumbprints” of candidates—the specific traits that set them apart, but the final hiring decision remains firmly in human hands. This collaborative approach ensures that technology enhances human intuition rather than replacing it.

Furthermore, the study suggests that the recruitment process with Generative AI’s value proposition should be bolstered by a “qualification guarantee.” By integrating verification checks and fraud detection, such as lip-sync and script-reading analysis, the recruitment process with Generative AI ensures that the person being interviewed is indeed the person being hired. This layer of accountability is crucial for companies that have been burned by deceptive practices in remote recruitment processes. Finally, the roadmap for the recruitment process with Generative AI involves a continuous feedback loop and technical evolution. Establishing a 24/7 technical support system and offering economical bundling packages will ensure long-term customer retention. As Indonesia continues its journey toward a digital-first economy, the integration of Gen-AI in recruitment will transition from a competitive advantage to an industry standard, provided it remains rooted in the practical, ethical, and cultural realities of the Indonesian workforce.

The findings of this feasibility study indicate a high potential for “the platform” within the Indonesian recruitment landscape. The discussion below integrates the observed results with the theoretical frameworks and existing literature.

### 5.1 Managerial implication

The results of the PESTEL analysis indicate that social and technological factors are the strongest drivers of adoption. With 83.6% of the Indonesian population familiar with AI, the “culture lag” identified in the results is a surmountable barrier rather than a structural failure. By reducing the “search friction” in a market with 7.27 million unemployed individuals, the recruitment Gen-AI platform directly addresses the labor supply–demand mismatch. The Porter’s Five Forces analysis reveals that while “Rivalry among Existing Competitors” is present, the majority of the global players lack localized linguistic capabilities. The result showing that the platform can conduct interviews in Bahasa Indonesia is a critical intangible resource. Furthermore, the high Barriers to Entry created by the US$60,991 R&D investment ensure that the platform maintains a competitive edge against low-cost, non-agentic alternatives.

The SWOT analysis identified robotic communication as a primary weakness. This is addressed in the Internal Risk Mitigation strategy, which suggests leveraging corporate capabilities to refine facial expression analysis. By linking the US$76,757 initial cost to specific R&D milestones, the study demonstrates how financial “Tangible Resources” are converted into technological “Strengths” that mitigate the Threats of professional resistance. The platform’s financial feasibility was evaluated through a five-year cost projection. For the initial development phase (Year 0/2025), the total capital requirement is estimated at US$76,757. A significant portion of this budget, approximately US$60,991 (79.5%), is dedicated to Research and Development (R&D). These costs represent the tangible resources within the resource-based view (RBV) framework, acting as the necessary investment to build the intangible assets (proprietary algorithms and brand equity) that will provide a competitive advantage.

One identified risk is insufficient Funding or High Operational Overheads. The study addresses this by aligning the cost projection with a tiered pricing model (Basic, Pro, and Max). This ensures that the Serviceable Obtainable Market (SOM) of US$1.75 billion is captured through a scalable revenue stream that offsets the high R&D burn rate identified in the results. Relating the costs back to the Introduction’s problem statement, the investment in AI automation is justified by the reduction in “time-to-hire.” While the initial setup cost of US$76,757 is substantial for a startup, the long-term reduction in HR department labor costs (which currently face a 44-day hiring cycle) offers a clear value proposition. This aligns with the PESTEL Economic factor, where Indonesian firms are increasingly seeking cost-efficient digital transformations to manage a growing labor force.

## 6 Conclusion and future research

In conclusion, this feasibility study confirms that the recruitment process with Generative AI is a highly viable and timely solution for the Indonesian recruitment market. By integrating Generative AI with video conferencing, the proposed system addresses the critical bottleneck of hiring efficiency, which currently burdens Indonesian firms with high costs and long selection periods. The study highlights that the technological readiness of the Indonesian workforce, combined with a supportive regulatory environment toward digital transformation, creates a significant opportunity for market entry with a projected Serviceable Obtainable Market (SOM) of US$1.75 billion.

However, the path to successful implementation requires a balanced approach between innovation and localization. To overcome “culture lag” and professional resistance, the recruitment process with Generative AI must be positioned as a supportive tool for HR professionals rather than a replacement. The feedback from Focus Group Discussions (FGD) serves as a strategic roadmap: prioritizing data security through robust firewalls, developing a linguistic engine capable of recognizing Indonesian dialects and slang, and offering customizable assessment metrics. These features will ensure the recruitment process with Generative AI is not only technologically superior but also culturally and operationally aligned with the specific needs of Indonesian enterprises.

Ultimately, the recruitment process with Generative AI stands to revolutionize talent acquisition in Indonesia by bridging the gap between high applicant volumes and the need for quality hires. By adopting the recommended tiered pricing models and ensuring a high standard of technical support and fraud detection, the project can maintain long-term financial sustainability. This research set out to evaluate the feasibility of integrating Generative AI into the Indonesian recruitment process. By synthesizing the strategic analysis and stakeholder feedback, the following conclusions are reached: Integration of PESTEL, Porter’s Five Forces, and SWOT frameworks confirms that the macro- and micro-environments in Indonesia are prepared for AI-driven recruitment. The platform aligns with national digital transformation goals and addresses the high time-to-hire (44 days) identified in the problem statement.

## Data Availability

The raw data supporting the conclusions of this article will be made available by the authors, without undue reservation.

## References

[ref1] Aguilar F. J. (1967). Scanning the Business Environment. United Kingdom: MacMillan.

[ref2] Ahmad S. Javed U. Sharma C. Siddiqui M. S. (2025). Green human resource management: analyzing sustainable practices and organizational impact through a Word2Vec approach. Green Technol. Sustain. 3:100224. doi: 10.1016/j.grets.2025.100224

[ref3] Ansoff H. I. (1958). A model for diversification. Manag. Sci. 4, 392–414. doi: 10.1287/mnsc.4.4.392

[ref4] Badan Pusat Statistik. (2025). Number and Percentage of Employment and Unemployment, 2025. Available online at: https://www.bps.go.id/idBusiness Indonesia https://www.bisnis.com/

[ref5] Badghish S. Soomro Y. A. (2024). Artificial intelligence adoption by SMES to achieve sustainable business performance: application of technology-organization-environment framework. Sustainability 16:1864. doi: 10.22495/jgrv13i4art8

[ref6] Baig M. I. Shuib L. Yadegaridehkordi E. (2019). Big data adoption: State of the art and research challenges. Inform. Process. Manag. 56:102095. doi: 10.1016/j.ipm.2019.102095

[ref9] Barney J. B. (2001). Resource-based theories of competitive advantage: a ten-year retrospective on the resource-based view. J. Manage. 27, 643–650. doi: 10.1016/S0149-2063(01)00115-5

[ref10] Bastida M. García A. V. Taín M. A. V. Araujo M. D. R. (2025). From automation to augmentation: human resource’s journey with artificial intelligence. J. Ind. Inf. Integr. 46:100872. doi: 10.1016/j.jii.2025.100872

[ref11] Benitez J. Arenas A. Castillo A. Esteves J. (2022). Impact of digital leadership capability on innovation performance: the role of platform digitization capability. Inf. Manag. 59:103590. doi: 10.1016/J.IM.2022.103590

[ref15] Brealey R. Myers S. Allen F. (2020). Principles of Corporate Finance. McGraw-Hill Education.

[ref17] Business Indonesia. (2025). Beyond the Slowdown: The Digital and Green Turn in Indonesian Employment. Available online at: https://business-indonesia.org/news/beyond-the-slowdown-the-digital-and-green-turn-in-indonesian-employment

[ref19] CareerPlug. (2024). 2024 Recruiting Metrics Report. CareerPlug. Available online at: https://www.careerplug.com/wp-content/uploads/2024/04/2024-Recruiting-Metrics-Report-1.pdf

[ref20] da Silva L. B. P. Soltovski R. Pontes J. Treinta F. T. Leitão P. Mosconi E. . (2022). Human resources management 4.0: literature review and trends. Comput. Ind. Eng. 168:108111. doi: 10.1016/j.cie.2022.108111

[ref22] Deloitte. (2024). 2024 Global Human Capital Trends: Thriving beyond boundaries: Human performance in a boundaryless world.

[ref23] Deloitte. (2025). 2025 HR technology marketplace predictions: New dynamics for working with humans and technology. Available online at: https://www.deloitte.com/global/en/issues/work/genz-millennial-survey.html

[ref24] Do H. Chu L. X. Shipton H. (2025). How and when AI-driven HRM promotes employee resilience and adaptive performance: a self-determination theory☆. J. Bus. Res. 192:115279. doi: 10.1016/j.jbusres.2025.115279

[ref25] Espegren Y. Hugosson M. (2025). HR analytics-as-practice: a systematic literature review. J. Organ. Eff. 12, 83–111.

[ref26] Faiz F. Le V. Masli E. K. (2024). Determinants of digital technology adoption in innovative SMEs. Journal of Innovation & Knowledge 9:100610. doi: 10.1016/j.jik.2024.100610

[ref29] Garcia R. L. F. Kwok L. (2025). Generative artificial intelligence in human resource management: a critical reflection on impacts, resilience, and roles. Int. J. Contemp. Hosp. Manag. 37, 3136–3158. doi: 10.1108/IJCHM-01-2025-0159

[ref31] Gupta S. Bostrom R. P. (2009). Technology-mediated learning: a comprehensive theoretical model. J. Assoc. Inf. Syst. 10, 101–114. doi: 10.17705/1jais.00207

[ref32] Headhunter Indonesia. (2025). Scribd. Available online at: https://www.scribd.com/document/820299355/Indonesia-s-Recruitment-Trend-Prediction-in-2025

[ref33] Hermann V. . (2024). Mental health status according to the dual-factor model in Swedish adolescents. PLoS ONE 19:e0299225. doi: 10.1371/journal.pone.0299225 38427682 PMC10906859

[ref34] Humphrey A. S. (2005). SWOT Analysis for Management Consulting. SRI Alumni Association Newsletter. Available online at: https://web.archive.org/web/20130104102543/http://www.sri.com/sites/default/files/brochures/dec-05.pdf

[ref35] Insight Sinergi Talenta. (2025). HR Tech Trends 2025: Apa Yang Harus Anda Terapkan Sekarang? Available online at: https://www.insightgroup.co.id/2025/02/26/hr-tech-trends-2025-apa-yang-harus-anda-terapkan-sekarang/

[ref40] Jayashree S. Reza M. N. H. Malarvizhi C. A. N. Gunasekaran A. Rauf M. A. (2022). Testing an adoption model for Industry 4.0 and sustainability: a Malaysian scenario. Sustain. Prod. Consump. 31, 351–364. doi: 10.1016/j.spc.2022.02.015

[ref41] JDIH Badan Pemeriksa Keuangan. (2008). *UU No. 20 Tahun 2008: Usaha Mikro, Kecil, dan Menengah*. Available online at: https://peraturan.bpk.go.id/Details/39653/uu-no-20-tahun-2008.

[ref42] JDIH Badan Pemeriksa Keuangan. (2019). *Peraturan Pemerintah (PP) Nomor 71 Tahun 2019: Penyelenggaraan Sistem dan Transaksi Elektronik*. Available online at: https://peraturan.bpk.go.id/Details/122030/pp-no-71-tahun-2019

[ref43] JDIH Badan Pemeriksa Keuangan. (2024). *Perubahan Kedua atas Undang-Undang Nomor 11 Tahun 2008 tentang Informasi dan Transaksi Elektronik*. Available online at: https://peraturan.bpk.go.id/details/274494/uu-no-1-tahun-2024

[ref44] JDIH Kemkomdigi. (2023). *Surat Edaran Menteri Komunikasi dan Informatika Nomor 9 Tahun 2023 tentang Etika Kecerdasan Artifisial*. Available online at: https://jdih.komdigi.go.id/produk_hukum/view/id/883/t/surat+edaran+menteri+komunikasi+dan+informatika+nomor+9+tahun+2023.

[ref47] Katadata Insight Center. (2025). *Kedaulatan AI untuk Memberdayakan Indonesia*. Available online at: https://databoks.katadata.co.id/en/publications/2025/01/23/kedaulatan-ai-untuk-memberdayakan-indonesia

[ref48] Kementerian Komunikasi dan Digital. (2024). *Membangun Ekosistem AI Di Indonesia Untuk 2030, Potensi Dan Tantangan*. Available online at: https://www.komdigi.go.id/berita/infrastruktur-digital/detail/membangun-ekosistem-ai-di-indonesia-untuk-2030-potensi-dan-tantangan

[ref49] Khan M. I. Parahyanti E. Hussain S. (2024). The role generative AI in human resource management: enhancing operational efficiency, decision-making, and addressing ethical challenges. Asian J. Logist. Manag. 3, 104–125. doi: 10.14710/ajlm.2024.24671.

[ref51] Kim M. Song J. (2014). Toward an integrated framework for innovation in service: a resource-based view and dynamic capabilities approach. Inf. Syst. Front. 17, 533–546. doi: 10.1007/s10796-014-9505-6

[ref53] Kotler P. Kartajaya H. Setiawan I. (2017). Marketing 4.0: Moving from Traditional to Digital. New York, United States: John Wiley & Sons.

[ref54] Kumar R. Chen X. (2024). Cultural dimensions of ai technology acceptance. Int. J. Inform. Manag. 65, 102–118. doi: 10.47134/converse.v2i1.4533

[ref56] Li B. Cheng Y. (2025). ChatGPT in human resource management: a systematic review of influential factors, processes, and outcomes. Heliyon 11:e44048. doi: 10.1016/j.heliyon.2025.e44048.

[ref57] LinkedIn. (2023). Global Talent Trends. Available online at https://business.linkedin.com/hire/global-talent-trends/archival/global-talent-trends-october-2023

[ref61] Millar C. Lockett M. Ladd T. (2018). Disruption: technology, innovation and society. Technol. Forecast. Soc. Change 129, 254–260. doi: 10.1016/J.TECHFORE.2017.10.020

[ref64] Mujianto N. Ramaditya M. Hartoyo N. Nurmalina R. Yusuf E. Z. (2025). The role of relationship quality mediation in traditional retail shop loyalty in the digital transformation era. Int. J. Electron. Mark. Retail. 16, 573–595. doi: 10.1504/ijemr.2025.148661

[ref65] Nishanti B. Hariharan C. Parthiban P. (2025). Unveiling the future of artificial intelligence in talent acquisition: a bibliometric analysis of emerging trends and future directions. Sustain. Futures 10:101410. doi: 10.1016/j.sftr.2025.101410.

[ref66] Pemerintah Daerah Kabupaten Sumbawa Barat. (2020). *Mengenal Usaha Mikro, Kecil Dan Menengah (Umkn)*. Available online at: https://peraturan.bpk.go.id/Details/277682/peraturan-bpk-no-1-tahun-2024

[ref67] Porter M. E. (1979). How competitive forces shape strategy. Harv. Bus. Rev. 57, 137–145. 18271320

[ref68] Ramadhan K. Nugroho W. S. (2025). Indonesia’s jobs boom is a story of quantity, not quality. East Asia Forum. Available online at: https://eastasiaforum.org/2025/07/17/indonesias-jobs-boom-is-a-story-of-quantity-not-quality/

[ref69] Ramaditya M. Effendi S. Burda A. (2023). Survival and human resource strategies of private higher education in facing an era of change: insight from Indonesia. Front. Educ. 8:1141123. doi: 10.3389/feduc.2023.1141123

[ref70] Ramaditya M. Maarif M. S. Affandi J. Sukmawati A. (2022). Reinventing talent management: how to maximize performance in higher education. Front. Educ. 7:929697. doi: 10.3389/feduc.2022.929697

[ref71] Ridhwan M. M. Suryahadi A. Rezki J. F. Andariesta D. T. (2023). The impact of COVID-19 on the labour market and the role of E-commerce development in developing countries: evidence from Indonesia. J. Asia-Pac. Econ. 30, 84–127. doi: 10.1080/13547860.2023.2195710

[ref72] Ritala P. Hurmelinna‐Laukkanen P. (2012). Incremental and radical innovation in coopetition—the role of absorptive capacity and appropriability. J. Product Innov. Manag. 30, 154–169. doi: 10.1111/j.1540-5885.2012.00956.x

[ref73] Satudata Ketenagakerjaan. (2022). *Perusahaan yang terdaftar di WLKP Online, Tw.IV Tahun 2022*. Available online at: https://satudata.kemnaker.go.id/data/kumpulan-data/1148

[ref75] Satudata Ketenagakerjaan. (2024). *Perusahaan yang terdaftar di WKLP Online, Tw.IV Tahun 2024*. Available online at: https://satudata.kemnaker.go.id/data/kumpulan-data/2449

[ref77] Setiyono F. (2022). A Journey to Indonesia’s 2030 Carbon Emission Reduction Target with Cloud and AI. Microsoft Source. Available online at: https://ejournal.ipinternasional.com/index.php/ijec/article/view/1337#:~:text=Journal%20of%20Government%20Science%2C%2012,of%202014%20concerning%

[ref78] Shahadat M. M. H. Nekmahmud M. Ebrahimi P. Fekete-Farkas M. (2023). Digital technology adoption in SMEs: what technological, environmental and organizational factors influence in emerging countries? Global Bus. Rev. 2, 4766–4774.

[ref79] Shchekina E. G. (2024). Digital reality as a new manifestation phenomena of border culture. Russ. Stud. Cult. Soc. 8.

[ref80] Shet S. V. Poddar T. Samuel F. W. Dwivedi Y. K. (2021). Examining the determinants of successful adoption of data analytics in human resource management—a framework for implications. J. Bus. Res. 131, 311–326. doi: 10.1016/j.jbusres.2021.03.054

[ref81] Su J. Yang W. (2023). Unlocking the power of ChatGPT: a framework for applying generative AI in education. ECNU Rev. Educ. 6, 355–366.

[ref82] Teece D. J. (2007). Explicating dynamic capabilities: the nature and micro foundations of (sustainable) enterprise performance. Strat. Manag. J. 28, 1319–1350. doi: 10.1002/smj.640

[ref83] Tomlinson B. Black R. W. Patterson D. J. Torrance A. W. (2024). The carbon emissions of writing and illustrating are lower for AI than for humans. Sci. Rep. 14:3732. doi: 10.1038/s41598-024-54271-x, 38355820 PMC10867074

[ref84] Wang Q. Li Y. Li R. (2024). Ecological footprints, carbon emissions, and energy transitions: the impact of artificial intelligence (AI). Humanit. Soc. Sci. Commun. 11:1043. doi: 10.1057/s41599-024-03520-5

